# Phylogeography of Influenza A(H3N2) Virus in Peru, 2010–2012

**DOI:** 10.3201/eid2108.150084

**Published:** 2015-08

**Authors:** Simon Pollett, Martha I. Nelson, Matthew Kasper, Yeny Tinoco, Mark Simons, Candice Romero, Marita Silva, Xudong Lin, Rebecca A. Halpin, Nadia Fedorova, Timothy B. Stockwell, David Wentworth, Edward C. Holmes, Daniel G. Bausch

**Affiliations:** University of California San Francisco, California, USA (S. Pollett);; United States Naval Medical Research Unit No. 6, Lima, Peru (S. Pollett, M. Kasper, Y. Tinoco, M. Simons, C. Romero, M. Silva, D.G. Bausch);; University of Sydney, Sydney, New South Wales, Australia (S. Pollett, E.C. Holmes);; National Institutes of Health, Bethesda, Maryland, USA (M.I. Nelson);; Arizona State University, Tempe, Arizona, USA (M.I. Nelson);; J. Craig Venter Institute, Rockville, Maryland, USA (X. Lin, R.A. Halpin, N. Fedorova, T.B. Stockwell, D. Wentworth);; Tulane School of Public Health and Tropical Medicine, New Orleans, Louisiana, USA (D.G. Bausch)

**Keywords:** Peru, H3N2, influenza virus, evolution, viruses, phylogeography, phylogeny, viral traffic, subtropical, tropical, gene pool, source population, surveillance, source–sink dynamic, source–sink model, interseasonal extinction, influenza A(H3N2) virus, human influenza, influenza

## Abstract

It remains unclear whether lineages of influenza A(H3N2) virus can persist in the tropics and seed temperate areas. We used viral gene sequence data sampled from Peru to test this source–sink model for a Latin American country. Viruses were obtained during 2010–2012 from influenza surveillance cohorts in Cusco, Tumbes, Puerto Maldonado, and Lima. Specimens positive for influenza A(H3N2) virus were randomly selected and underwent hemagglutinin sequencing and phylogeographic analyses. Analysis of 389 hemagglutinin sequences from Peru and 2,192 global sequences demonstrated interseasonal extinction of Peruvian lineages. Extensive mixing occurred with global clades, but some spatial structure was observed at all sites; this structure was weakest in Lima and Puerto Maldonado, indicating that these locations may experience greater viral traffic. The broad diversity and co-circulation of many simultaneous lineages of H3N2 virus in Peru suggests that this country should not be overlooked as a potential source for novel pandemic strains.

## Abstract 2

Peru did not fit the source–sink model for the global spread of this virus.

Worldwide, influenza virus causes substantial illness and death and considerable public health costs (*1*). Like other countries, Peru experiences a significant number of influenza cases (*2*,*3*). The epidemiology of influenza virus in tropical and low- to middle-income countries and the role they play in global influenza ecology remains unclear (*4*). One outstanding question is whether a global source–sink dynamic exists. In the source–sink model, countries have putative tropical sources of influenza characterized by year-round (or multiannual) transmission, local persistence of influenza lineages, and relatively high genetic diversity. Then, it is postulated, that influenza lineages migrate and seed seasonal epidemics in cooler temperate regions, where they experience interseasonal extinction (*5*). Determining if and where this source–sink dynamic exists is of major importance because the results could guide targeted influenza surveillance for vaccine recommendations, pandemic planning, and prediction of novel strains (*4*,*6*).

Most analyses of whether a global source population exists have focused on East and Southeast Asia, in part because several pandemic and seasonal epidemics appear to have originated in those areas (*7*–*11*). Because of the lower availability of local influenza sequence data from tropical Latin America, relatively little is known about the possible role that region plays in global influenza dynamics (*12*). Nonmolecular epidemiologic studies have hinted at climate-driven patterns of influenza virus spread in South America; for example, diffusion of influenza activity from tropical to temperate areas has been noted in Brazil (*13*). Peru’s diverse climates make it an ideal location to test aspects of the source–sink model in Latin America, particularly because some tropical areas in Peru are known to experience year-round influenza activity (*14*). In recent years, prospective community-based influenza-like illness (ILI) surveillance cohorts were established in multiple regions of Peru, providing a unique opportunity to examine the epidemiology of human influenza virus (*15*).

Our study objectives were to determine whether 1) a source–sink influenza dynamic exists within Peru, including the existence of genetically diverse hubs and virus lineage persistence between seasons; 2) Peru could act as a global source for influenza virus lineages that could seed temperate regions; and 3) influenza virus is circulating within Peru in a closed system. We also sought to compare the spatial dynamics of influenza A(H3N2) virus across the 4 climatically and demographically diverse Peruvian sites.

We based our analysis on human influenza A(H3N2) virus because, over a long-term scale, it is the best represented lineage in sequence databases, and it has caused regular seasonal influenza epidemics in both hemispheres, including in Latin America (*16*,*17*). Although much attention has been paid to the study of pandemic influenza A(H1N1)pdm09 virus (*18*), H3N2 virus remains a significant cause of influenza in Peru, is a dominant seasonal influenza A virus subtype in other regions of the world, and causes substantial illness and death in Peru and beyond. A key aspect of this study is that we obtained samples from diverse ecologies and populations, including viruses from large urban and semirural locations and diverse altitudes and climates, and the distance between study sites was sufficient to allow spatial analysis. In addition, the prospective cohort studies involved continuous, active, year-round surveillance that enabled capture of any interseasonal strains.

## Materials and Methods

### Study Setting, Enrolment Criteria, and Field Procedures

In 2009, the United States Navy Medical Research Unit No. 6 (NAMRU-6), the Centers for Disease Control and Prevention (CDC), and the Peruvian Ministry of Health established a community-based prospective ILI cohort (Proyecto Influenza) in 4 ecologically distinct regions of Peru. Sites were chosen to represent the diverse ecologies, climates, and population structure in Peru. Lima, on the central desert coast, is Peru’s capital and largest city and a transport hub for the rest of the nation. Lima has a population of 8,348,400 persons and a temperate climate with little rain (*19*). Puerto Maldonado, in the southern Amazon Basin, has a population of 89,500 persons. The city has high annual rainfall and a warm, humid climate year-round (*19*). Cusco is a high-altitude (3,200 meters) city in the southern Andes Mountains. This southern highlands city has a population of 420,030 persons (*19*). Tumbes is a northern equatorial coastal city of 157,760 persons (*19*).

Enrollment criteria and field procedures were as described elsewhere (*15*). In brief, during 2010–2012, households were selected from each study site by using a computer-based randomization process. An adult head and all residents of the household were eligible for enrollment. Participants were assessed 3 times per week for the development of ILI. For children <5 years of age, ILI was defined as sudden onset of fever (>38°C) and cough, sore throat, or coryza. For persons >5 years of age, ILI was defined as sudden onset of fever (>38°C) with cough, sore throat, or both. We administered a household enrolment form in which sociodemographic and risk factor data were collected. Nasal and throat swab samples for virus identification were obtained from persons with signs meeting the ILI case definition; a rapid influenza test was performed so that immediate medical referral could be made if necessary.

### Ethical Approval

The NAMRU-6 Institutional Review Board approved the study. Informed written consent was obtained at the time of enrolment from each adult participant and from a parent or guardian of children. NAMRU-6 participation was under protocol NMRCD.2009.005, which is in compliance with all applicable US federal regulations governing the protection of human subjects.

### Detection of Influenza Virus in Nasal or Throat Swab Specimens

Nucleic acid was extracted from nasal and throat swab specimens in universal transport media by using the QIAamp Viral RNA Isolation Kit (QIAGEN, Valencia, CA, USA). Reverse transcription PCR (RT-PCR) for influenza detection, including subtype, was performed by using primers and probes from the CDC Human Influenza Virus Real-Time RT-PCR Diagnostic Panel (Influenza Reagent Resource, CDC, Atlanta, GA, USA). Original respiratory samples were then stored at −80°C at NAMRU-6 Peru.

### Identification of Sequences for Phylogenomic Analyses and Generation of Sequence Data

Over the study period, we randomly selected 100 H3N2 virus–positive (RT-PCR cycle threshold <29) specimens from each study site (400 total). Original respiratory specimens were sent (at −80°C) from NAMRU-6 Peru to the J. Craig Venter Institute (Rockville, MD, USA) for extraction and hemagglutinin (HA) gene sequencing. GenBank accession numbers for the consensus sequences are available in Technical Appendix Table. Viral RNA was isolated by using the ZR 96 Viral RNA Kit (Zymo Research Corporation, Irvine, CA, USA). The influenza A virus genomic RNA segments were simultaneously amplified from purified RNA (3 μL) by using a multisegment RT-PCR strategy (*20*,*21*). Amplicons were sequenced by using the Nextera DNA Sample Preparation Kit Library construction and the Illumina MiSeq version 2 platform (both from Illumina, Inc., San Diego, CA, USA) or the Ion Xpress Plus Fragment Library Kit and the Ion Torrent PGM platform (both from Thermo Fisher Scientific, Waltham, MA, USA). The sequence reads were sorted by barcode and trimmed, and chimeric influenza virus sequences and noninfluenza sequences were removed. The next-generation sequencing reads were then mapped to the best matching reference virus by using the CLC Bio Assembly Cell 3.0 program clc_ref_assemble_long (http://www.clcbio.com/products/clc-assembly-cell/) (*22*). At loci where next-generation sequencing platforms agreed on a variation (as compared with the reference sequence), the reference sequence was updated to reflect the difference. A final mapping of all next-generation sequences to the updated reference sequences was then performed.

### Collation of Background Sequence Data, Alignment, and Evolutionary Model Selection

Global background H3N2 HA sequences were obtained from the National Institute of Allergy and Infectious Disease Influenza Research Database (IRD; http://www.fludb.org/brc/home.spg?decorator=influenza) (*23*) and the Global Initiative on Sharing Avian Influenza Data EpiFlu Database (http://platform.gisaid.org/epi3/frontend#f989c). Sequences for viruses obtained during January 2004–August 2013 from the following regions were sampled (nos. in parentheses indicate no. of sequences): South America, excluding Peru (193); Australia, New Zealand, and Oceania, excluding Hawaii (259); East and Southeast Asia (374); Middle East/Central Asia, including Russia (110); Europe (235); Central America and the Caribbean (116); Mexico (27); Canada (234); the United States, including Hawaii (549); and Africa (79). In addition, 16 sequences for strains collected in Peru during 2006–2013 were obtained through IRD or the EpiFlu Database. A total of 2,192 background sequences were selected (Technical Appendix Tables 2–4).

To improve phylogenetic resolution, only complete or near-complete HA sequences (containing at least the entire HA1 region) were included. For geographic regions with an abundance of full HA1 sequences in GenBank (e.g., Asia, United States), intermittent sequences were manually selected from a list sorted by country in the IRD. For underrepresented geographic regions (e.g., Africa, South America), all available full HA1 sequences were included to overcome ascertainment bias. Accession numbers (GenBank and EpiFlu Database) for these comparator sequences are shown in Technical Appendix Tables 2–4.

Untranslated regions were trimmed, and duplicate sequences were removed, resulting in a final dataset of 2,581 sequences 1,639–1,700 nt in length; 1 partial sequence was 1,324 nt long. A second dataset of 389 sequences (1,700 nt long) was constructed for viruses from Peru. All sequences were aligned before inspection by using the MUSCLE algorithm in MEGA5.2 and hand-edited for final correction (*24*). A best-fit model of nucleotide substitution (general time-reversible with a gamma-distributed rate variation among sites and a proportion of invariant sites) was selected by using jModelTest2 software (*25*).

### Global Phylogenetic Analysis

A maximum-likelihood tree of all 2,581 H3 sequences was inferred by using RAxML software version 7.26 (*26*). Statistical robustness was tested by nonparametric bootstrap resampling analysis (500 replicates). Inferred maximum-likelihood trees were viewed and annotated by using FigTree software (http://tree.bio.ed.ac.uk/software/figtree/).

### Bayesian Analyses of Peruvian Sequences

We analyzed 389 HA time-stamped sequences (i.e., labeled with the time of sampling to the nearest day) for viruses from Peru by using the Bayesian Markov chain Monte Carlo method in BEAST (*27*); the results enabled inference of the time-scale of the viruses’ epidemiologic histories. For this analysis, we selected a Bayesian skyline demographic model was selected and, assuming a strict molecular clock rate (under a uniform prior), we selected the Hasegawa-Kishino-Yano nucleotide substitution model with a discrete-gamma distribution in place of other, more complex models that likely overparameterized the data. The analysis was run by using a 500,000,000-step Markov chain, sampling every 50,000 states. A 10% burn-in was removed, and statistical convergence was determined by parameter values with effective sample size values >200. The posterior distribution of trees was summarized as the maximum clade credibility tree, as generated by using TreeAnnotator version 1.75 (http://beast.bio.edu.ac.uk/TreeAnnotator/) and visualized by using FigTree (http://tree.bio.ed.ac.uk/software/figtree/).

For viruses from Peru, the posterior distribution of HA trees from BEAST was also used to assess the strength of geographic clustering in the data by using the phylogeny-trait association test available in the Bayesian Tip-association Significance testing package (*28*). For this analysis, each sequence was given a geographic code reflecting its place of origin. The overall statistical significance of geographic clustering of all Peruvian sequences by location was determined by calculating observed and expected association index and parsimony score statistics for the entire Peruvian sequence dataset, where the null hypothesis is that clustering by geographic location is not more than that expected by chance. In addition, the maximum clade statistic was used to compare the strength of clustering at each location by calculating the expected and observed mean clade size from each of the 4 study locations. A significance level of p<0.05 was used in all cases.

## Results

Of the 400 H3N2 PCR–positive specimens selected from the NAMRU-6 repository, 389 HA segments were successfully sequenced (Technical Appendix Table 1). The distribution of successfully sequenced H3N2 HA genes by year and location relative to other co-circulating influenza virus subtypes in the study period is presented in Table 1. Well-distributed sampling in all sites for all years was impossible because of differences in specimen quality and because overall H3N2 virus activity in the cohorts was considerably less overall during 2011–2012 than in 2010, partly due to the dominance of influenza B virus in 2012. Thus, the sampling was skewed toward 2010 and toward fewer sequences for Cusco and Puerto Maldonado in 2012 and Tumbes in 2011.

**Table 1:** Distribution of sequenced influenza A(H3N2) virus strains, compared with all confirmed cases of influenza and influenza-like illness, Peru, 2010–2012

Year, location	No. sequenced influenza A(H3N2) strains*	No. other strains or illnesses				
		All H3N2	Influenza A(H1N1) pdm09	Influenza B	Influenza illness	Influenza-like illness
2010						
All	227	414	138	306	858	1,716
Lima	41	95	38	96	229	458
Cusco	31	42	63	74	179	358
Tumbes	92	155	25	83	263	526
Puerto Maldonado	63	122	12	53	187	374
2011						
All	105	219	36	16	271	542
Lima	13	35	6	1	42	84
Cusco	65	101	2	0	103	206
Tumbes	2	17	11	14	42	84
Puerto Maldonado	25	66	17	1	84	168
2012						
All	57	87	57	233	377	754
Lima	27	45	29	48	122	244
Cusco	0	7	7	74	88	176
Tumbes	28	42	18	18	78	156
Puerto Maldonado	2	38	3	93	134	268
2010–2012						
All	389	1,485	462	1110	3057	6,114

*Strains sequenced during this phylogeographic study of influenza A(H3N2) virus in Peru.

**Figure 1:**
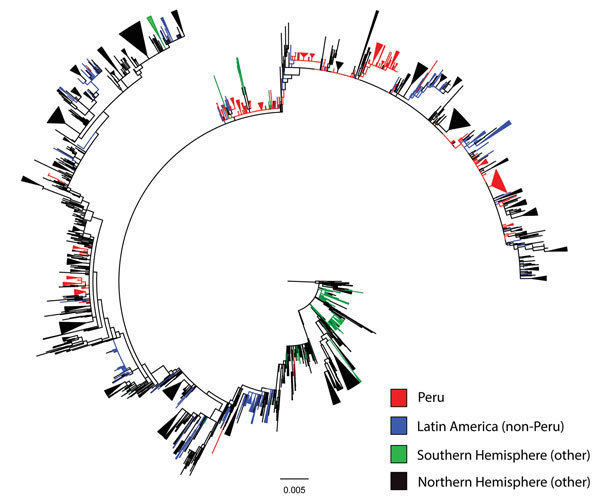
Maximum-likelihood phylogeny of hemagglutinin sequences of influenza A(H3N2) viruses from Peru and other global locations, rooted with the oldest available sequence (A/Hong Kong/CUHK52390/2004). Scale bar indicates number of nucleotide substitutions per site.

**Figure 2:**
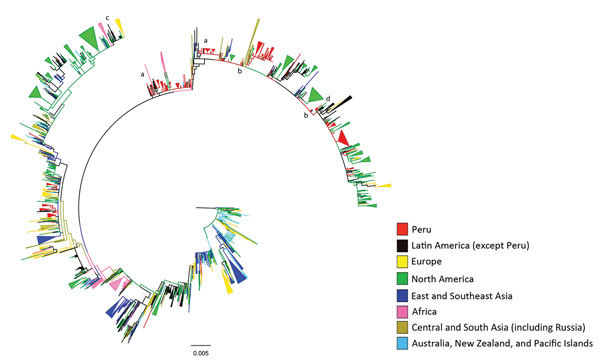
Maximum likelihood phylogeny of hemagglutinin sequences of influenza A(H3N2) viruses from Peru and other global locations, rooted on the oldest available sequence (A/Hong Kong/CUHK52390/2004). Clades containing strains from Peru and a neighboring country are indicated by the letters a–c, designating Bolivia (a), Chile (b), and Ecuador (c). The letter d indicates phylogenetic evidence of migration from Peru to North America, with onward transmission after seeding that region. Scale bar indicates number of nucleotide substitutions per site.

Phylogenetic analysis of the 389 study sequences for viruses from Peru and 2,192 global HA sequences revealed extensive geographic mixing (Figures 1, 2; fully labeled tree in the Technical Appendix Figure). Perhaps the most notable observation from this analysis was the interseasonal extinction of virus clades from Peru in all regions of the country, even in a tropical region where molecularly confirmed year-round influenza transmission has been noted (*14*). In addition, the phylogeny showed extensive global mixing of H3N2 viruses, with co-circulation of clades from Peru with those from all Northern and Southern Hemisphere regions, including in countries in Latin and North America, Africa, Europe, Central Asia, and East and Southeast Asia. In one instance, onward transmission of virus was noted after migration from Peru to the United States (Figure 2, section d).

Viruses from each study location in Peru formed weak to moderately supported clades with sequences for viruses from other localities (bootstrap values were usually <70% but occasionally >80%), reflecting a relative lack of phylogenetic resolution in the data at this scale (online Technical Appendix Figure). In contrast, smaller but often better supported clades (frequently with bootstrap values >70%) containing H3 virus sequences from multiple locations in Peru were observed (online Technical Appendix Figure).

Closer examination of the phylogenetic analysis of sequences for viruses from Latin America showed evidence for the presence of weakly supported sublineages consisting predominantly of strains from Peru but also containing strains from Chile and Bolivia (Figure 2); this finding is indicative of viral traffic between these border-sharing countries. Analysis of clustering with strains from Ecuador was limited by a paucity of sequences, but evidence of strongly supported clustering with strains from Peru was found (Figure 2). In addition, strains from Peru fell into some weakly supported multinational sublineages containing strains from Brazil, Venezuela, Paraguay, Nicaragua, Colombia, Argentina, and Mexico, which suggests H3N2 viral traffic throughout the Americas (online Technical Appendix Figure).

**Figure 3:**
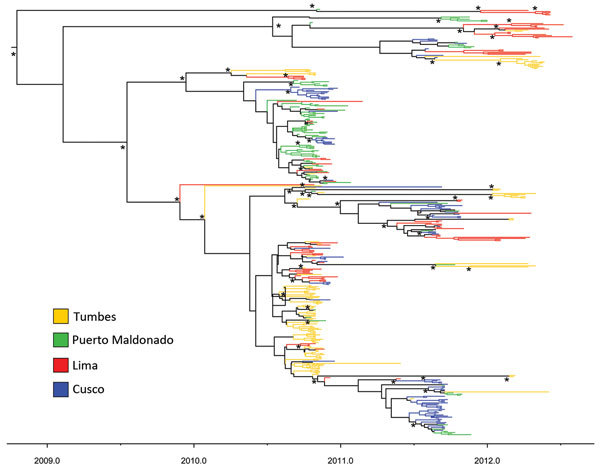
Time-scaled maximum clade credibility phylogeny of hemagglutinin sequences for influenza A(H3N2) viruses from 4 locations in Peru. *Indicates posterior probabilities >0.9. Scale bar refers to year of sampling to indicate time of sampling for each virus.

Analyzed separately, the maximum clade credibility tree (Figure 3) for strains from Peru showed substantial HA diversity each year; many clades co-circulated at each location. The smaller-sized locations of Tumbes, Puerto Maldonado, and Cusco had a wide range of co-circulating clades, similar to those of larger travel hubs, such as Lima (Table 2). This analysis also showed a short time to most common recent ancestor (mean 3.8 y, 95% highest posterior density 3.1–4.6 y), as has been shown for most other studied localities (*5*,*29*). A similarly short mean time to most recent common ancestor (1.6 y, 95% highest posterior density 1.1–2.1 y) was obtained for 2010, the most sampled year, providing the most precise single-season estimate.

**Table 2:** Number of circulating influenza A(H3N2) virus clades, Peru, 2010–2012*

Location	No. clades circulating, by year		
	2010	2011	2012
Lima	8	6	5
Puerto Maldonado	6	4	0
Cusco	4	9	0
Tumbes	13	1	5

*Data are derived from the phylogenetic tree in Figure 3.

To determine the phylogeographic structure in the data, we performed phylogeny-trait association tests (Table 3). For strains from Peru, the results confirmed a stronger spatial clustering of sequences at all sites than would be expected by chance alone (p<0.01), but the results also showed clear evidence of some viral traffic among sampling locations, as noted in the phylogenetic analysis. Furthermore, the maximum clade statistic was significant (p = 0.009) in all 4 study sites, reflecting predominantly local evolution in these localities. Differences in the observed and expected maximum clade values tentatively suggested that Lima exhibited the least structure (i.e., most mixing; difference of 5.50) and Tumbes the strongest spatial structure (difference of 10.33) (Table 3).

**Table 3:** Results of phylogeny-trait association testing for influenza A(H3N2) viruses in Peru, 2010–2012*

Location	Association index (95% CI)†				Parsimony scores (95% CI)†				Mean maximum clade size (95% CI)‡			
	Observed	Expected	p value§		Observed	Expected	p value§		Observed	Expected	p value¶	Difference#
All	8.53 (7.25–9.81)	33.02 (31.52–34.56)	<0.001		73.72 (70.00–77.00)	211.00 (205.65–217.36)	<0.001		–	–		–
Lima	–	–			–	–			8.04 (6.0−10.0)	2.6 (2.18−3.16)	0.009	5.44
Cusco	–	–			–	–			12.4 (12.0−15.0)	2.82 (2.36−3.44)	0.009	9.58
Puerto Maldonado	–	–			–	–			8.2 (6.0−14.0)	2.7 (2.28−3.45)	0.009	5.50
Tumbes	–	–			–	–			13.68 (10.0−22.0)	3.35 (2.76−4.99)	0.009	10.33

*Results were determined by a Bayesian analysis of phylogeographic structure. p values correspond to the proportion of trees from the null distribution equal to, or more extreme than, the median posterior of the statistic.  †Association index and parsimony scores only determined for all locations combined. ‡Maximum clade size statistics only determined for each specific location. §p<0.001 confirms a stronger observed spatial clustering of sequences from Peru at all sites than would be expected by chance alone. ¶p = 0.009 reflects predominantly local evolution in the 4 locations. #Difference between observed and expected clade size.

## Discussion

Our phylogenetic analysis showed that the level of international H3N2 viral traffic was high and that mixing of Peruvian HA sequences with those from multiple regions of the world was rapid and widespread (Figures 1, 2). These findings support a continuous H3N2 gene flow in and out of Peru rather than a local closed system in which viruses evolve entirely within the country. Mixing of viruses between all study sites in Peru and other countries may also suggest gene flow in and out of Peruvian locations outside the main air-transport hub of Lima. However, such a conclusion comes with a strong caveat because we may not have sampled all Lima source lineages that seed peripheral locations in the country. Of note, we found evidence of H3N2 virus migration between Peru and its neighbors, although this conclusion was limited by a relative paucity of sequences from these other Latin American countries.

At each study site in Peru, we found multiple co-circulating clades of influenza virus that regularly underwent extinction (Figures 1, 2), suggesting that much of the genetic diversity of viruses in Peru results from global lineages that pass through the country, rather than from local evolution associated with long-term local persistence. In particular, all sampled strains, even those from tropical Peruvian sites like Tumbes and Puerto Maldonado, underwent extinction rather than persisted over time, thus regularly halting local evolution of imported influenza viruses. That the time to most common recent ancestor of the whole sample (mean 3.8 y) was much shorter than the known history of H3N2 virus in Peru is also consistent with the idea that the influenza virus gene pool in Peru is being frequently replenished from other regions.

Our findings are consistent with those of studies in countries with temperate regions, such as Australia, New Zealand, and countries in North America, which showed regular introduction of new H3N2 virus lineages and seeding of local seasonal epidemics rather than the interseasonal persistence of lineages (*29*–*31*). Such studies have similarly revealed that the genetic diversity of seasonal influenza in temperate locales primarily results from the on-going introduction of genetically divergent lineages during seasonal epidemics (*5*,*30*–*32*).

In contrast, interseasonal persistence of H3N2 influenza virus has been documented in subtropical and tropical locations like Hong Kong and Southeast Asia (*7*,*8*,*10*). A more recent study has shown evidence for multiyear pandemic influenza A(H1N1)pdm09 strain persistence in tropical areas of western Africa that are relatively isolated (*33*). In contrast, an analysis of H3N2 virus persistence over a 15-year period in subtropical China did not demonstrate interseasonal persistence, and the sample size in that study was much larger than that in our study (*9*).

Our findings did not offer support to a source–sink dynamic within Peru, and they also indicate that Peru is an unlikely common tropical source of persistent lineages that seed other countries in Latin America or the rest of the world. Instead, our findings are more consistent with a shifting metapopulation model of H3N2 virus, such that the virus may pass through any region for a variable amount of time rather than perpetually circulating in fixed locations in the tropics and consistently seeding temperate regions each year (*11*,*34*). Such a shifting metapopulation model may also explain why some studies show apparent persistence in some tropical and subtropical locations over certain years and others do not (*7*–*9*,*33*). This model is also compatible with the existence of temporary source populations in locations throughout the world. Indeed, we provide some phylogenetic evidence that Peru may occasionally, but not consistently, act as a temporary source, spreading virus from Peru to another country, from which onward transmission continues (Figure 2, section d).

H3 virus sequences for viruses from Peru also exhibited some clustering by sampling location, a finding consistent with semilocalized seasonal H3N2 virus epidemics in each region of Peru (Figure 3), although with migration between localities. Such semilocalized epidemics have been observed in other areas (*29*). These data also provided some evidence for weaker spatial clustering in Lima compared with other localities. This evidence is not surprising because Lima has the largest population and, thus, movement of humans around, in, or out of the city would generally be expected to be greater than in other areas. In this context it is perhaps surprising that Puerto Maldonado, the least populous site, had a similar strength of spatial clustering. This locality has been characterized by rapid population growth, likely due to widespread mining and associated activities (*35*). Hence, it is possible that frequent human movement in and out of this location is creating more diffusion of influenza virus. In addition, the true population of this area may be considerably higher than suggested by official statistics.

These findings have implications for public health practice in Peru and Latin America. For example, they suggest that future novel strains of influenza virus may enter Peru at multiple locations rather than just through its major air-transport hub (Lima) (*36*). Moreover, the rapid diffusion of influenza virus throughout Peru, even in the more remote regions, also serves as a potent reminder of how quickly influenza virus can disseminate. We identified Lima and Puerto Maldonado as possible diffusion hubs for influenza virus; perhaps both cities could be prioritized for heightened influenza surveillance if a novel influenza subtype is introduced into Peru.

Although Peru does not appear to be a global source population for influenza viruses, the diversity and co-circulation of many simultaneous lineages of H3N2 virus in the country means that it should not be overlooked as a potential source for novel pandemic strains, particularly given that there is some evidence of high-risk animal farming practices and low biosecurity in this country (*37*). Similarly, the rapid, widespread, and unpredictable migration of global strains into Peru and widespread global mixing shown in this study emphasize that vaccine recommendations in either hemisphere need to be based on well distributed, widespread global H3N2 virus sampling from as many sentinel laboratories as possible (*6*).

Technical Appendix Fully annotated maximum-likelihood phylogeny of HA sequences from Peru and other regions of the world and accession numbers and sequences for various strains used in this study.

## References

[1] Fineberg HV. Pandemic preparedness and response–lessons from the H1N1 influenza of 2009. N Engl J Med. 2014;370:1335–42. 10.1056/NEJMra1208802 24693893

[2] Forshey BM, Laguna-Torres VA, Vilcarromero S, Bazan I, Rocha C, Morrison AC, Epidemiology of influenza-like illness in the Amazon Basin of Peru, 2008–2009. Influenza Other Respir Viruses. 2010;4:235–43. 10.1111/j.1750-2659.2010.00139.x PMC5964548 20836798

[3] Tinoco Y, Razuri H, Ortiz EJ, Gomez J, Widdowson MA, Uyeki T, Preliminary population-based epidemiological and clinical data on 2009 pandemic H1N1 influenza A (pH1N1) from Lima, Peru. Influenza Other Respir Viruses. 2009;3:253–6. 10.1111/j.1750-2659.2009.00111.x PMC4940804 19903205

[4] Viboud C, Alonso WJ, Simonsen L. Influenza in tropical regions. PLoS Med. 2006;3:e89. 10.1371/journal.pmed.0030089 16509764 PMC1391975

[5] Rambaut A, Pybus OG, Nelson MI, Viboud C, Taubenberger JK, Holmes EC. The genomic and epidemiological dynamics of human influenza A virus. Nature. 2008;453:615–9. 10.1038/nature06945 18418375 PMC2441973

[6] Russell CA, Jones TC, Barr IG, Cox NJ, Garten RJ, Gregory V, Influenza vaccine strain selection and recent studies on the global migration of seasonal influenza viruses. Vaccine. 2008;26(Suppl 4):D31–4. 19230156 10.1016/j.vaccine.2008.07.078

[7] Bahl J, Nelson MI, Chan KH, Chen R, Vijaykrishna D, Halpin RA, Temporally structured metapopulation dynamics and persistence of influenza A H3N2 virus in humans. Proc Natl Acad Sci U S A. 2011;108:19359–64. 10.1073/pnas.1109314108 22084096 PMC3228450

[8] Le MQ, Lam HM, Cuong VD, Lam TT, Halpin RA, Wentworth DE, Migration and persistence of human influenza A viruses, Vietnam, 2001–2008. Emerg Infect Dis. 2013;19:1756–65. 10.3201/eid1911.130349 24188643 PMC3837676

[9] Cheng X, Tan Y, He M, Lam TT, Lu X, Viboud C, Epidemiological dynamics and phylogeography of influenza virus in southern China. J Infect Dis. 2013;207:106–14. 10.1093/infdis/jis526 22930808 PMC3523792

[10] Tang JW, Ngai KL, Lam WY, Chan PK. Seasonality of influenza A(H3N2) virus: a Hong Kong perspective (1997–2006). PLoS ONE. 2008;3:e2768. 10.1371/journal.pone.0002768 18648550 PMC2481298

[11] Russell CA, Jones TC, Barr IG, Cox NJ, Garten RJ, Gregory V, The global circulation of seasonal influenza A (H3N2) viruses. Science. 2008;320:340–6. 10.1126/science.1154137 18420927

[12] Nelson MI, Balmaseda A, Kuan G, Saborio S, Lin X, Halpin RA, The evolutionary dynamics of influenza A and B viruses in the tropical city of Managua, Nicaragua. Virology. 2014;462–463:81–90. 10.1016/j.virol.2014.05.025 24959982 PMC4175446

[13] Alonso WJ, Viboud C, Simonsen L, Hirano EW, Daufenbach LZ, Miller MA. Seasonality of influenza in Brazil: a traveling wave from the Amazon to the subtropics. Am J Epidemiol. 2007;165:1434–42. 10.1093/aje/kwm012 17369609

[14] Laguna-Torres VA, Gomez J, Ocana V, Aguilar P, Saldarriaga T, Chavez E, Influenza-like illness sentinel surveillance in Peru. PLoS ONE. 2009;4:e6118. 10.1371/journal.pone.0006118 19568433 PMC2700970

[15] Razuri H, Romero C, Tinoco Y, Guezala MC, Ortiz E, Silva M, Population-based active surveillance cohort studies for influenza: lessons from Peru. Bull World Health Organ. 2012;90:318–20 . 22511830 10.2471/BLT.11.097808 PMC3324870

[16] Cox NJ, Subbarao K. Global epidemiology of influenza: past and present. Annu Rev Med. 2000;51:407–21. 10.1146/annurev.med.51.1.407 10774473

[17] The pink book. Influenza: epidemiology and prevention of vaccine-preventable diseases. Influenza virus [cited 2013 Nov 1]. http://www.cdc.gov/vaccines/pubs/pinkbook/flu.html#flu

[18] Cheng VC, To KK, Tse H, Hung IF, Yuen KY. Two years after pandemic influenza A/2009/H1N1: what have we learned? Clin Microbiol Rev. 2012;25:223–63. 10.1128/CMR.05012-11 22491771 PMC3346300

[19] Instituto Nacional de Estadistica e informatica [cited 2013 Oct 1]. http://www.inei.gob.pe

[20] Zhou B, Donnelly ME, Scholes DT, St George K, Hatta M, Kawaoka Y, Single-reaction genomic amplification accelerates sequencing and vaccine production for classical and swine origin human influenza a viruses. J Virol. 2009;83:10309–13. 10.1128/JVI.01109-09 19605485 PMC2748056

[21] Zhou B, Wentworth DE. Influenza A virus molecular virology techniques. Methods Mol Biol. 2012;865:175–92. 22528160 10.1007/978-1-61779-621-0_11

[22] White paper on reference assembly in CLC Assembly Cell 3.0. May 10, 2010 [cited 2013 Nov 1]. http://www.clcbio.com/wp-content/uploads/2012/09/white_paper_on_reference_assembly_on_the_CLC_Assembly_Cell.pdf

[23] Squires RB, Noronha J, Hunt V, Garcia-Sastre A, Macken C, Baumgarth N, Influenza Research Database: an integrated bioinformatics resource for influenza research and surveillance. Influenza Other Respir Viruses. 2012;6:404–16. 10.1111/j.1750-2659.2011.00331.x PMC3345175 22260278

[24] Tamura K, Peterson D, Peterson N, Stecher G, Nei M, Kumar S. MEGA5: molecular evolutionary genetics analysis using maximum likelihood, evolutionary distance, and maximum parsimony methods. Mol Biol Evol. 2011;28:2731–9. 10.1093/molbev/msr121 21546353 PMC3203626

[25] Darriba D, Taboada GL, Doallo R, Posada D. jModelTest 2: more models, new heuristics and parallel computing. Nat Methods. 2012;9:772. 10.1038/nmeth.2109 22847109 PMC4594756

[26] Stamatakis A, Ludwig T, Meier H. RAxML-III: a fast program for maximum likelihood–based inference of large phylogenetic trees. Bioinformatics. 2005;21:456–63. 10.1093/bioinformatics/bti191 15608047

[27] Drummond AJ, Suchard MA, Xie D, Rambaut A. Bayesian phylogenetics with BEAUti and the BEAST 1.7. Mol Biol Evol. 2012;29:1969–73. 10.1093/molbev/mss075 22367748 PMC3408070

[28] Parker J, Rambaut A, Pybus OG. Correlating viral phenotypes with phylogeny: accounting for phylogenetic uncertainty. Infect Genet Evol. 2008;8:239–46. 10.1016/j.meegid.2007.08.001 17921073

[29] Nelson MI, Simonsen L, Viboud C, Miller MA, Taylor J, George KS, Stochastic processes are key determinants of short-term evolution in influenza a virus. PLoS Pathog. 2006;2:e125. 10.1371/journal.ppat.0020125 17140286 PMC1665651

[30] Holmes EC, Ghedin E, Miller N, Taylor J, Bao Y, St George K, Whole-genome analysis of human influenza A virus reveals multiple persistent lineages and reassortment among recent H3N2 viruses. PLoS Biol. 2005;3:e300. 10.1371/journal.pbio.0030300 16026181 PMC1180517

[31] Nelson MI, Simonsen L, Viboud C, Miller MA, Holmes EC. Phylogenetic analysis reveals the global migration of seasonal influenza A viruses. PLoS Pathog. 2007;3:1220–8. 10.1371/journal.ppat.0030131 17941707 PMC2323296

[32] Lavenu A, Leruez-Ville M, Chaix ML, Boelle PY, Rogez S, Freymuth F, Detailed analysis of the genetic evolution of influenza virus during the course of an epidemic. Epidemiol Infect. 2006;134:514–20. 10.1017/S0950268805005686 16316493 PMC2870435

[33] Nelson MI, Njouom R, Viboud C, Niang MN, Kadjo H, Ampofo W, Multiyear persistence of 2 pandemic A/H1N1 influenza virus lineages in west Africa. J Infect Dis. 2014;210:121–5. 10.1093/infdis/jiu047 24446525 PMC4162001

[34] Lemey P, Rambaut A, Bedford T, Faria N, Bielejec F, Baele G, Unifying viral genetics and human transportation data to predict the global transmission dynamics of human influenza H3N2. PLoS Pathog. 2014;10:e1003932. 10.1371/journal.ppat.1003932 24586153 PMC3930559

[35] Asner GP, Llactayo W, Tupayachi R, Luna ER. Elevated rates of gold mining in the Amazon revealed through high-resolution monitoring. Proc Natl Acad Sci U S A. 2013;110:18454–9. 10.1073/pnas.1318271110 24167281 PMC3832012

[36] Chowell G, Viboud C, Munayco CV, Gomez J, Simonsen L, Miller MA, Spatial and temporal characteristics of the 2009 A/H1N1 influenza pandemic in Peru. PLoS ONE. 2011;6:e21287. 10.1371/journal.pone.0021287 21712984 PMC3119673

[37] McCune S, Arriola CS, Gilman RH, Romero MA, Ayvar V, Cama VA, Interspecies interactions and potential Influenza A virus risk in small swine farms in Peru. BMC Infect Dis. 2012;12:58. 10.1186/1471-2334-12-58 22420542 PMC3364844

