# Supplementary material for: Phylogeography of Influenza A(H3N2) Virus in Peru, 2010–2012
Source: Emerg Infect Dis. 2015 Aug;21(8):1330–8. doi: 10.3201/eid2108.150084 (PMC4517729; doi:10.3201/eid2108.150084)
Supplement: Technical Appendix — Fully annotated maximum-likelihood phylogeny of HA sequences from Peru and other regions of the world and accession numbers and sequences for various strains used in this study. [file 15-0084-Techapp-s1.pdf]

Article DOI: http://dx.doi.org/10.3201/eid2108.150084

Phylogeography of Influenza A(H3N2) Virus in Peru, 2010–2012

Technical Appendix

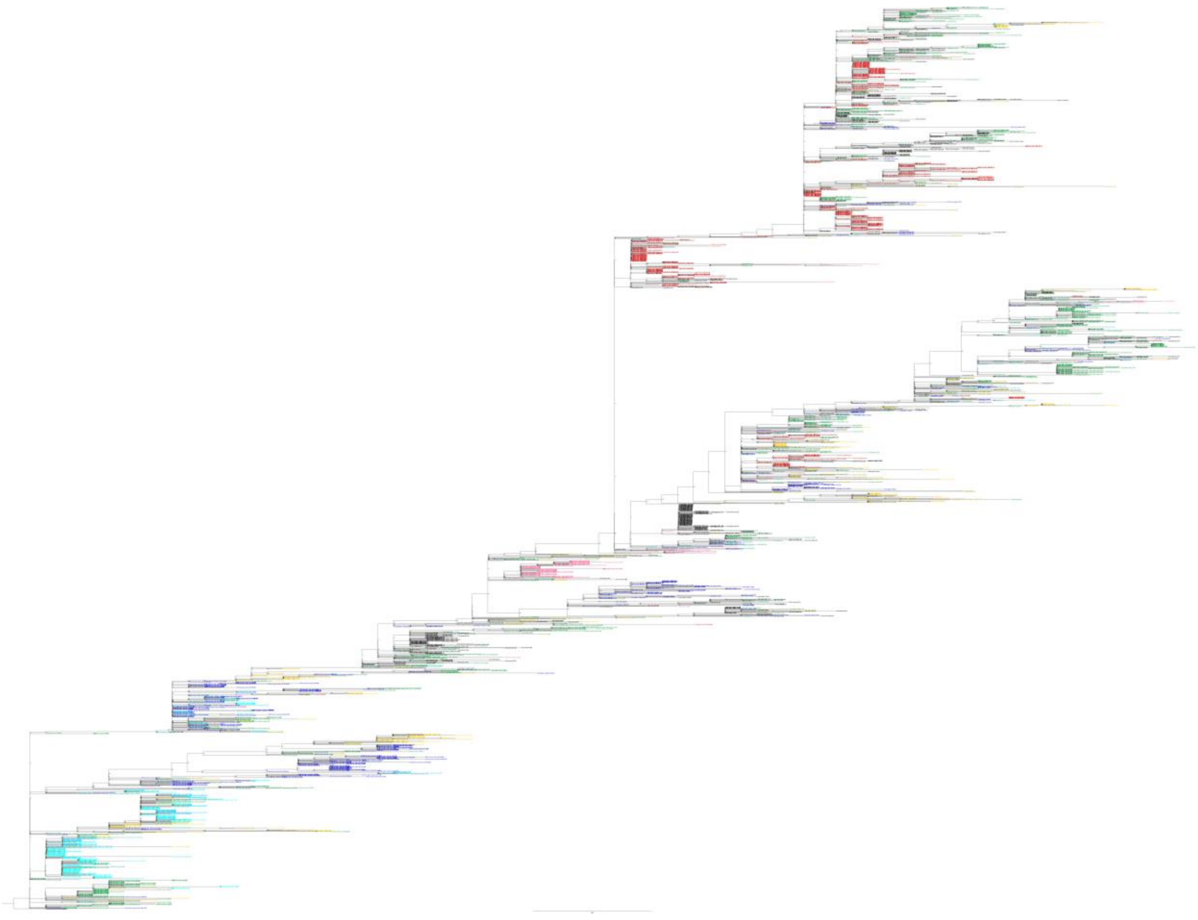

Technical Appendix Figure. Maximum-likelihood phylogeny of hemagglutinin sequences from Peru and other regions of the world, temporally rooted (A/Hong Kong/CUHK52390/2004). All tip labels are included and nodes are annotated by bootstrap (http://wwwnc.cdc.gov/eid/images/15-0084 TechApp-F.jpg). Fine detail may be viewed using the zoom function in PDF viewer software. Colored tip labels refer to global regions in the context of Peruvian taxa: Peru (red), Asia including East Asia and SE Asia (deep blue), Europe (yellow), North America including USA (excluding Hawaii), Mexico and Canada (green), Caribbean and Central/South America excluding Peru (black), Africa (pink), Australia, New Zealand and Oceania (including Hawaii) (light blue), Middle East/Central Asia/South Asia and Russia (brown). Nodes and branches have not been assigned a geographic location.

Technical Appendix Table 1. Accession numbers of Peruvian strains sequenced from this study

Accession nos.						
CY160276.1	CY160768.1	CY161240.1	CY161736.1	CY162224.1	CY162704.1	CY163184.1
CY160281.1	CY160776.1	CY161248.1	CY161744.1	CY162232.1	CY162712.1	CY163192.1
CY160288.1	CY160784.1	CY161256.1	CY161752.1	CY162240.1	CY162720.1	CY163200.1
CY160296.1	CY160792.1	CY161264.1	CY161760.1	CY162248.1	CY162728.1	CY163208.1
CY160304.1	CY160800.1	CY161272.1	CY161768.1	CY162256.1	CY162736.1	CY163216.1
CY160312.1	CY160808.1	CY161280.1	CY161776.1	CY162264.1	CY162744.1	CY163224.1
CY160320.1	CY160816.1	CY161288.1	CY161784.1	CY162272.1	CY162752.1	CY163240.1
CY160328.1	CY160824.1	CY161296.1	CY161792.1	CY162280.1	CY162760.1	CY163248.1
CY160336.1	CY160832.1	CY161304.1	CY161800.1	CY162288.1	CY162768.1	CY163256.1
CY160344.1	CY160840.1	CY161312.1	CY161808.1	CY162296.1	CY162776.1	CY163264.1
CY160352.1	CY160848.1	CY161320.1	CY161816.1	CY162304.1	CY162784.1	CY163272.1
CY160360.1	CY160856.1	CY161328.1	CY161824.1	CY162312.1	CY162792.1	CY163280.1
CY160368.1	CY160864.1	CY161336.1	CY161832.1	CY162320.1	CY162800.1	CY163288.1
CY160376.1	CY160872.1	CY161344.1	CY161840.1	CY162328.1	CY162808.1	CY163296.1
CY160384.1	CY160880.1	CY161352.1	CY161848.1	CY162336.1	CY162816.1	CY163304.1
CY160392.1	CY160888.1	CY161360.1	CY161856.1	CY162344.1	CY162824.1	CY163312.1
CY160400.1	CY160896.1	CY161368.1	CY161864.1	CY162352.1	CY162832.1	CY163320.1
CY160408.1	CY160904.1	CY161376.1	CY161872.1	CY162360.1	CY162840.1	CY163328.1
CY160416.1	CY160912.1	CY161384.1	CY161880.1	CY162368.1	CY162848.1	CY163336.1
CY160424.1	CY160920.1	CY161392.1	CY161888.1	CY162376.1	CY162856.1	CY163344.1
CY160432.1	CY160928.1	CY161400.1	CY161896.1	CY162384.1	CY162864.1	CY163352.1
CY160440.1	CY160936.1	CY161408.1	CY161904.1	CY162392.1	CY162872.1	CY163360.1
CY160448.1	CY160944.1	CY161416.1	CY161912.1	CY162400.1	CY162880.1	CY163368.1
CY160456.1	CY160952.1	CY161424.1	CY161920.1	CY162408.1	CY162888.1	CY163376.1
CY160464.1	CY160960.1	CY161432.1	CY161928.1	CY162416.1	CY162896.1	CY163384.1
CY160472.1	CY160968.1	CY161440.1	CY161936.1	CY162424.1	CY162904.1	CY163392.1
CY160480.1	CY160976.1	CY161448.1	CY161944.1	CY162432.1	CY162912.1	CY163400.1
CY160488.1	CY160984.1	CY161456.1	CY161952.1	CY162440.1	CY162920.1	CY161720.1
CY160496.1	CY160992.1	CY161464.1	CY161960.1	CY162448.1	CY162928.1	CY161728.1
CY160504.1	CY161000.1	CY161472.1	CY161968.1	CY162456.1	CY162936.1	CY162216.1
CY160512.1	CY161008.1	CY161480.1	CY161976.1	CY162464.1	CY162944.1	CY162696.1
CY160520.1	CY161016.1	CY161488.1	CY161984.1	CY162472.1	CY162952.1	CY163176.1
CY160528.1	CY161024.1	CY161496.1	CY161992.1	CY162480.1	CY162960.1	
CY160536.1	CY161032.1	CY161504.1	CY162000.1	CY162488.1	CY162968.1	
CY160544.1	CY161040.1	CY161512.1	CY162008.1	CY162496.1	CY162976.1	
CY160560.1	CY161048.1	CY161520.1	CY162016.1	CY162504.1	CY162984.1	
CY160568.1	CY161056.1	CY161528.1	CY162032.1	CY162512.1	CY162992.1	
CY160576.1	CY161064.1	CY161536.1	CY162040.1	CY162520.1	CY163000.1	
CY160584.1	CY161072.1	CY161544.1	CY162048.1	CY162528.1	CY163008.1	
CY160592.1	CY161080.1	CY161552.1	CY162056.1	CY162536.1	CY163016.1	
CY160600.1	CY161088.1	CY161560.1	CY162064.1	CY162544.1	CY163024.1	
CY160608.1	CY161096.1	CY161568.1	CY162072.1	CY162552.1	CY163032.1	
CY160616.1	CY161104.1	CY161576.1	CY162080.1	CY162560.1	CY163040.1	
CY160624.1	CY161112.1	CY161584.1	CY162088.1	CY162568.1	CY163048.1	
CY160632.1	CY161120.1	CY161592.1	CY162096.1	CY162576.1	CY163056.1	
CY160648.1	CY161128.1	CY161600.1	CY162104.1	CY162584.1	CY163064.1	
CY160656.1	CY161136.1	CY161608.1	CY162112.1	CY162592.1	CY163072.1	
CY160664.1	CY161144.1	CY161616.1	CY162120.1	CY162600.1	CY163080.1	
CY160672.1	CY161152.1	CY161624.1	CY162128.1	CY162608.1	CY163088.1	
CY160680.1	CY161160.1	CY161632.1	CY162136.1	CY162616.1	CY163096.1	
CY160688.1	CY161168.1	CY161640.1	CY162144.1	CY162624.1	CY163104.1	
CY160696.1	CY161176.1	CY161656.1	CY162152.1	CY162632.1	CY163112.1	
CY160704.1	CY161184.1	CY161664.1	CY162160.1	CY162640.1	CY163120.1	
CY160720.1	CY161192.1	CY161672.1	CY162168.1	CY162648.1	CY163128.1	
CY160728.1	CY161200.1	CY161680.1	CY162176.1	CY162656.1	CY163136.1	
CY160736.1	CY161208.1	CY161688.1	CY162184.1	CY162664.1	CY163144.1	
CY160744.1	CY161216.1	CY161696.1	CY162192.1	CY162672.1	CY163152.1	
CY160752.1	CY161224.1	CY161704.1	CY162200.1	CY162680.1	CY163160.1	
CY160760.1	CY161232.1	CY161712.1	CY162208.1	CY162688.1	CY163168.1	

Technical Appendix Table 2. Background GenBank sequences for the Pacific Islands, Americas, and Europe

Pacific Islands	Eastern United States	Western United States	Canada and Alaska, USA	Mexico, Central America, and Caribbean	Europe	South America
CY130191	KF789944	KC893099	KC882748	CY088899	HQ880599	HM628693
CY141203	KC892790	KC892863	KF789947	CY074843	JF327387	HM628694
CY141204	KC892482	KF790270	KC535500	CY074747	JF327386	CY093407
CY147307	KF789560	KF790275	KC892853	CY070951	JX518887	CY093415
CY147308	KC883350	KC882759	KF790212	CY088891	CY114501	JN872427
CY147309	KC892934	KF790514	KF790407	CY073869	CY114509	JN872405
CY147310	KC892860	KC882781	KC535486	CY074779	CY114421	JN872406
KC535402	KF790032	KC882493	KC892629	CY098081	CY114553	JN872407
KC535444	KC883362	CY141180	KF790118	CY088995	CY093391	JN872408
KC882467	KC892856	KC513483	KC882754	CY074795	CY093399	JN872409
KC882647	KF790482	KC513484	KF790088	CY070943	JX913067	JN872412
KC882762	KC883000	KC513479	KC892601	CY074763	JX913019	JN872414
KC882769	KC535302	KC513482	KF790391	CY074731	JX913027	KF142477
KC883166	KC883367	KC513477	KC882787	CY088915	JX913035	JN872420
KC883193	KC892174	KC892796	KC882453	CY092305	JX913059	CY070144
KC892157	KF790361	KC882483	KF790378	CY074915	JX913011	JN872423
KC892204	KC883054	KF790153	KF789569	CY088851	JX913072	JN872421
KC892382	KC892724	KC883183	KC883393	CY070959	JX913074	JN872418
KC892397	KC882657	KC893075	KC883090	CY074739	JX913079	JN872417
KC892459	KC883275	KC535428	KC883402	CY088907	JX913003	JN872415
KC892661	KC883253	KF790455	KC883438	CY074931	JX913043	JN872413
KC892764	KC882583	KF790509	KC892536	CY074699	JX913051	KC291190
KC892772	KC882690	KF790517	KC892914	CY088971	JX978770	JN872429
KC892786	KC883179	KC882902	KC892420	CY074771	KC488834	JN872416
KC892959	KF789968	KF789546	KC892668	CY088843	KC488809	JN872410
KC893140	KC892899	KF789906	KF790196	CY088979	JX978740	KF142476
KC893145	KC883449	KF789535	KF790216	CY092313	KC488843	JN872422
	KC535396	KC535295	KF790184	CY074827	KC488827	JN872424
	KC882692	KC882908	KF790195	CY074875	KC488826	JN872411
	KC893147	KF789627	KF790238	CY074891	KC135510	HM628692
	KC892260	KC883270	KF790255	CY098073	JQ988033	JN872425
	CY134637	KC892437	KF789796	CY088955	KC488837 *	JN872426
	CY134638	KC882651	CY134659	CY088987	CY093567	JN872428
	CY134648	KC883338	CY147301	CY088867	CY093575	JN872419
	CY134649	KC892364	KF598716	CY074803	KC135496	KC291191
	KC892544	KF789840	KF598717	CY092289	KC135504	JX679214
	CY141185	KC883292	KF598718	CY088939	KC135500	EU716428
	CY141186	KC892367	KF598719	CY074675	KC135508	CY121632
	CY141187	KC883093	KF598720	CY074707	JN940429	EU716426
	CY141188	KC892829	KF598721	CY089003	JN940431	EU716429
	CY141189	KF789739	KF598722	CY074835	KC135506	
	CY141190	KC892985	KF598723	CY070935	KC135502	
	CY141191	KC892850	KF598724	CY074867	KC135498	
	CY141192	KC882777	KF598725	CY074899	JN940427	
	CY141193	KC882784	KF598702	CY074819	CY114538	
	CY141194	KC892253	KF598728	CY088883	CY114558	
	CY141195	CY120885	KF598703	CY074923	JX978743	
	CY141196	KC892971	KF598729	CY089011	KC488812	
	CY141197	KC892677	KF598704	CY088931	JX978737	
	CY141199	KC892655	KF598730	CY074691	KC488807	
	CY141200	KC882482	KF598705	CY089027	CY114533	
	CY147291	KC892638	KF598706	CY074859	JX978734	
	KF789983	CY147299	KF598732	CY088875	JX978761	
	KF790197	CY147305	KF598707	CY088774	KC488820	
	KC892953	KC892641	KF598733	CY074907	JX978746	
	KC882724	KC892490	KF598708	CY088859	KC488815	
	KC882462	KC892747	KF598709	CY088790	JX978764	
	KF789585	KF790448	KF598710	CY088947	JX978767	
	KF790050	KF790054	KF598711	CY074851	KC488831 *	
	KF142471	KF789927	KF598712	CY088782	KC488823	
	KC883203	KF789534	KF598713	CY074811	JX978776	
	KF790330	CY091581	KF598714	CY093117	CY114548	
	KC893107	CY092281	CY111004	CY092297	KC135512	
	KC893166	CY092265	KF551068	CY088923	JQ988045	
	KF789614	CY092273	KC526207	CY070927	CY110774	
	KF790532	KF199854	CY111005	CY098065	CY110775	
	KF789660	CY068081	KC526208	CY074883	CY110776	
	KC882545	KC882579	CY111007	CY089019	KC488817	
	KC883261	KC882584	CY111006	CY074715		

Pacific Islands	Eastern United States	Western United States	Canada and Alaska, USA	Mexico, Central America, and Caribbean	Europe	South America
	KC882523	KF790512	KC526210	CY088963		
	KC883427	KC883258	CY111008	CY074683		
	KC892576	KC893012	KF551069	CY103791		
	KC892723	KF789646	KC526211	CY074755		
	KC882447	KC882644	CY111011	CY074787		
	KC892236	KF789567	KC526212	CY074723		
	KC892696	KC892376	CY111009	CY093375		
	KF789664	KC883221	KC526213	CY093471		
	KF789667	KC883246	CY111010	CY093479		
	CY141209	KC892462	KC526214	CY093503		
	CY141210	KC892968	KF551070	CY093511		
	CY141211	KC892227	CY111013	CY093519		
	KF790187	KC893064	CY110990	CY093527		
	KF790524	KC892859	CY110991	CY093535		
	KF789674	KF790214	CY110992	CY093543		
	KC893110	KF789637	CY110993	CY093551		
	KC893183	KF789799	CY110994	CY093559		
	KC893180	KF789828	KF551072	CY093447		
	KF790135	KC892812	CY110995	CY093423		
	KC892456	KF790454	CY110996	CY093431		
	KF789949	KC535447	CY110997	CY093439		
	KC535419	KF790503	CY110998			
	KC883129	KC882764	CY110999			
	KF790039	KC883336	KF551074			
	KC882652	KF789550	CY111000			
	KC883156	KF790036	KF551075			
	KF790362	KC883408	CY111001			
	KC882606	KC882609	CY111002			
	KC892889	KC883086	CY111003			
	KC883137	KC892361	KF551067			
	KC882836	KC892635	KF551076			
	KC892978	KC892600	KF551077			
	KC892573	KC892608	KF551078			
	KC892719	KC892845	KC526204			
	KC892896	KC892879	KC526205			
	KC892758	KC892876	KC526206			
	CY070967	KC892874	JQ658890			
	CY072214	KC892871	JQ658925			
	CY134640	KC892206	JQ658889			
	CY134641	KC892221	JQ658891			
	CY134643	KC892392	JQ658892			
	CY134644	KC892224	JQ658901			
	CY134645	KC893067	JQ658895			
	CY134646	KF790242	JQ658896			
	CY134647	KF789590	JQ658898			
	CY134650	KF789759	JQ658899			
	CY134651	CY141215	JQ658897			
	CY134652	CY141216	JQ658903			
	CY134653	KF790329	JQ658900			
	CY134654	KC882914	JQ658902			
	CY134661	KC892370	JQ658921			
	CY134686	KC883443	JQ658907			
	CY141220	KF790461	JQ658904			
	CY141221	KC892552	JQ658923			
	CY141222	KF790473	JQ658913			
	CY141223	KC882900	JQ658914			
	CY141224	KF790457	JQ658908			
	CY141225	KC892508	JQ658909			
	CY141226	KF789731	JQ658910			
	CY141227	KC892822	JQ658911			
	CY141228	KF790019	JQ658912			
	CY141229	KC883415	JQ658927			
	CY141230	KC892209	JQ658926			
	CY141231	KF790023	JQ658920			
	CY141232	KC883095	JQ658918			
	CY141234	KC892954	JQ658919			
	CY141235	KF789767	JQ658915			
	CY141239	KC892751	JQ658916			
	CY141240	KF790323	JQ658917			
	CY141241	KF790384	JQ658893			
	CY141242	KC893072	JQ658894			
	CY141243	KF789656	JQ658905			

Pacific Islands	Eastern United States	Western United States	Canada and Alaska, USA	Mexico, Central America, and Caribbean	Europe	South America
	CY141244	KF789977	JQ658906			
	CY141245	KC882772	JQ658924			
	CY141246	KC892358	JQ658888			
	CY141247	KC892549	KF598738			
	CY141248	KC883084	KF598743			
	KF789982	KC892931	KF761498			
	KF790180	KC882793	KF761499			
	KF790282	KC893047	KF761500			
	KF790236	KC892198	KF761501			
	KF790277	KC892212	KF761503			
	KF790258	KF790432	KF761505			
	KF790278	KC892960	KF761506			
	KF789822	KF789752	KF761507			
	KF790419	KF790077	KF761508			
	KF790394	KC535372	KF761509			
	CY084334	KC535405	KF761510			
	KC882595	KC892815	KF761511			
	KC893087	KC882736	KF761512			
	KF790201	KC882775	KF761513			
	KF790138	KC882917	KF685747			
	KF789613	KF790331				
	KC883240	KC883394				
	KF789728	KF789544				
	KF789842	KF789847				
	KC892471	KC883407				
	KC892555	KC892352				
	KC892193	KC892355				
	KC892279	KC883099				
	KC882431	KC892379				
	KC892156	KC892388				
	KC892177	KC892444				
	KC892149	KC892802				
	KC882577	KC892504				
	KC892976	KC892618				
	KC892524	KC892885				
	KF789582	KC892882				
	KF789770	KC892675				
	CY141249	CY120883				
	CY141250	CY141276				
	CY141251	CY141277				
		CY141278				
		CY141279				
		CY141280				
		CY141281				
		CY141282				
		KC892241				
		KC892665				
		KF790001				
		KF790167				
		KF790516				
		KF789753				
		KF789818				
		KF789826				

Technical Appendix Table 3. GenBank background sequences Asia, Australia, New Zealand, and Africa

Middle East and Central Asia	Australia and New Zealand	China, including Hong Kong	Singapore	Africa	Northeast Asia
KC865653	CY090869	CY091827	JX437710	CY062337	AB796432
KC865611	CY090877	CY091837	JX437711	CY062338	CY120889
KC865637		CY091841	KF014202	CY062339	CY130200
KC865649		CY091843	KF014130	CY062340	CY147295
KC865651		CY091845	JX437712	CY062341	CY147296
KC865613		CY099953	JX437713	CY062342	CY147298
KC865621		HQ664924	JX437714	CY062343	CY147300
KC865619		HQ664914	JX437715	CY062344	CY147303
KC709818		HQ664931	JX437716	CY062345	CY147294
KC865609		CY050136	JX437717	CY062346	CY147302
CY116636		CY050138	KF014203	CY062349	CY147311
CY116638		CY050102	JX437718	CY062350	HQ703350
CY116640		CY050104	JX437719	CY062351	HQ703352
KC865647		CY050106	KF014204	CY062352	
KC865655		CY050108	KF014205	JQ396181	
KC865657		CY050110	KF014131	JQ396183	
KC865659		CY050111	KF014206	JQ396184	
KC865661		CY050115	KF014132	JQ396185	
KC865663		CY050123	KF014133	KF451872	
KC865615		CY050125	KF014207	KF451873	
KC865617		CY050127	KF014208	KF451875	
KC865623		CY050128	JX437720	KF451876	
KC865625		CY050089	KF014209	KF451877	
KC865627		CY050090	KF014134	KF451878	
KC865629		CY050091	KF014135	KF451880	
KC865631		CY050093	KF014210	KF451881	
KC865633		CY050095	KF014136	KF451882	
KC865635		CY050097	KF014211	KF451883	
KC865639		JQ220541	KF014137	KF451884	
KC865641		JN256732	KF014212	KF451885	
KC865643		CY106912	KF014138	JQ396182	
KC865645		JN256733	KF014213	KC999473	
		CY115776	KF014214	KC999477	
		CY106920	KF014139	KC999474	
		JN256736	KF014215	KC999475	
		CY106944	KF014140	KC999476	
		CY115800	KF014141		
		JN256738	KF014216		
		CY106960	KF014217		
		JN256739	KF014142		
		CY115816	KF014218		
		CY106968	JX437721		
		JN256740	KF014143		
		CY115824	KF014219		
		JN256741	KF014220		
		CY106984	KF014144		
		JN256742	JX437842		
		CY106992	KF014145		
			KF014221		
			KF014146		
			KF014222		
			KF014147		
			JX437722		
			KF014223		
			KF014224		
			KF014148		
			KF014225		
			KF014149		
			KF014226		
			KF014150		
			KF014227		
			KF014151		
			KF014228		
			KF014152		
			CY124151		
			CY124153		
			CY124155		
			CY124157		

Middle East and Central Asia	Australia and New Zealand	China, including Hong Kong	Singapore	Africa	Northeast Asia
			CY124159		
			CY124161		
			CY124163		
			CY124165		
			CY124167		
			CY124169		
			CY124171		
			CY124173		
			CY124175		
			CY124177		
			CY124179		
			CY124181		
			CY124183		
			CY124185		
			CY124187		
			CY124189		
			CY124191		
			CY124193		
			CY124197		
			CY124199		
			CY124201		
			CY124203		
			CY124205		
			CY124207		
			CY124209		
			CY124211		
			CY124213		
			CY124215		
			CY124217		
			CY124221		
			CY124223		
			CY124225		
			CY124229		
			CY124233		
			CY124237		
			CY124239		
			CY124241		
			CY124243		
			CY124245		
			CY124247		
			CY124249		
			CY124251		
			CY124253		
			CY124255		
			CY124257		
			CY124259		
			CY124261		
			CY124263		
			CY124265		
			CY124267		
			CY124269		
			CY124271		
			CY124273		
			CY124275		
			CY124277		
			CY124279		
			CY124281		
			CY124283		
			CY124285		
			CY124287		
			CY124291		
			CY100091		
			CY100093		
			CY100075		
			CY100095		
			CY100077		
			CY100097		
			CY100099		
			CY124293		
			CY100079		
			CY100081		
			CY100101		
			CY100103		

Middle East and Central Asia	Australia and New Zealand	China, including Hong Kong	Singapore	Africa	Northeast Asia
			CY100105		
			CY100107		
			CY100109		
			CY124295		
			CY100111		
			CY100113		
			CY100115		
			CY100117		
			CY100119		
			CY124297		
			CY124299		
			CY124301		
			KF014233		
			KF014160		
			KF014161		
			KF014234		
			KF014235		
			KF014162		
			JX437831		
			KF014163		
			JX437832		
			KF014164		
			KF014165		
			KF014236		
			KF014237		
			KF014166		
			JX437833		
			KF014167		
			KF014168		
			JX437834		
			JX437835		
			KF014169		
			KF014238		
			KF014170		
			JX437836		
			KF014171		
			KF014239		
			KF014172		
			KF014173		
			KF014240		
			KF014174		
			JX437837		
			KF014175		
			KF014241		
			KF014242		
			KF014176		
			KF014177		
			JX437838		
			KF014178		
			KF014243		
			KF014244		
			KF014179		
			KF014180		
			KF014245		
			KF014181		
			KF014246		
			KF014247		
			KF014182		
			KF014183		
			KF014248		
			KF014249		
			KF014184		
			KF014185		
			KF014250		
			KF014186		
			KF014251		
			JX437839		
			KF014187		
			KF014252		
			KF014188		
			KF014189		
			KF014253		
			KF014254		

Middle East and Central Asia	Australia and New Zealand	China, including Hong Kong	Singapore	Africa	Northeast Asia
			KF014190		
			KF014191		
			KF014255		
			JX437840		
			KF014192		
			KF014193		
			KF014256		
			KF014194		
			KF014257		
			KF014195		
			KF014258		
			KF014196		
			JX437841		
			KF432083		
			CY124303		
			CY124305		
			CY124307		
			CY124309		
			CY124313		
			CY124315		
			CY124317		
			CY124319		
			CY124321		
			CY124323		
			CY124325		
			CY124327		
			CY124329		
			CY124331		
			CY124333		
			CY124335		
			CY124337		
			CY124339		
			CY124343		
			CY124345		
			CY124347		
			CY124349		
			CY124351		
			CY124353		
			CY124355		
			CY124357		
			CY124359		
			CY124361		
			CY124363		
			CY124365		
			CY124367		
			CY124369		
			CY124371		
			CY124373		
			CY124375		
			CY124377		
			CY124379		
			CY124381		
			CY124383		
			CY100087		
			CY100089		
			CY124387		
			CY124389		
			CY124391		
			CY124393		
			CY124395		
			CY124399		
			CY124401		
			CY124403		
			CY100125		
			CY100127		
			CY124407		
			CY124409		
			CY124411		
			CY124413		
			CY124417		
			CY124419		
			CY124423		
			CY124429		

Middle East and Central Asia	Australia and New Zealand	China, including Hong Kong	Singapore	Africa	Northeast Asia
			CY124431		
			CY124433		
			CY124435		
			CY124437		
			CY124439		
			CY124441		
			CY124443		
			CY124445		
			CY124447		
			CY124449		
			CY124453		
			CY124463		
			CY124465		
			CY124467		
			CY124469		
			CY124471		
			CY124473		
			CY124475		
			CY124477		
			CY124481		
			CY124483		
			CY124485		
			CY124487		
			CY124489		
			CY124491		
			CY124493		
			CY124499		
			CY124501		

Technical Appendix Table 4. GISAID Reference HA H3N2 Sequences for South America*

Strain name (includes country)	Strain ID	Originating laboratory	Submitting laboratory
A/Paraguay/06/2013	EPI_ISL_149684	Central Laboratory of Public Health	CDC
A/Bolivia/902/2013	EPI_ISL_146007	CENETROP	CDC
A/Santiago/47142/2013	EPI_ISL_145682	Instituto de Salud Publica de Chile	CDC
A/Uruguay/322/2013	EPI_ISL_145680	Departamento de Laboratorio de Salud Publica	CDC
A/French Guiana/1118/2013	EPI_ISL_145679	National Influenza Center French Guiana and French Indies	CDC
A/Uruguay/396/2013	EPI_ISL_145678	Departamento de Laboratorio de Salud Publica	CDC
A/Brazil/3873/2013	EPI_ISL_145677	Instituto Adolfo Lutz	CDC
A/Valparaiso/34097/2013	EPI_ISL_145676	Instituto de Salud Publica de Chile	CDC
A/Santiago/46150/2013	EPI_ISL_145671	Instituto de Salud Publica de Chile	CDC
A/Peru/140/2013	EPI_ISL_145670	NAMRU-6	CDC
A/Santiago/36541/2013	EPI_ISL_145642	Instituto de Salud Publica de Chile	CDC
A/Santiago/35652/2013	EPI_ISL_145641	Instituto de Salud Publica de Chile	CDC
A/Venezuela/05/2013	EPI_ISL_145515	Instituto Nacional de Higiene "Rafael Rangel"	CDC
A/Brazil/265/2013	EPI_ISL_145511	Oswaldo Cruz Foundation - Ministry of Health	CDC
A/Ecuador/440/2013	EPI_ISL_145510	NAMRU-6	CDC
A/Brazil/0328/2013	EPI_ISL_145500	Instituto Adolfo Lutz	CDC
A/Brazil/0289/2013	EPI_ISL_145126	Oswaldo Cruz Foundation - Ministry of Health	CDC
A/Argentina/555/2013	EPI_ISL_145122	Instituto Nacional de Enfermedades Infecciosas	CDC
A/Argentina/45/2013	EPI_ISL_145121	Instituto Nacional de Enfermedades Infecciosas	CDC
A/Argentina/433/2013	EPI_ISL_145114	Instituto Nacional de Enfermedades Infecciosas	CDC
A/Argentina/206/2013	EPI_ISL_145113	Instituto Nacional de Enfermedades Infecciosas	CDC
A/Brazil/0328/2013	EPI_ISL_144304	Instituto Adolfo Lutz	CDC
A/Santiago/20181/2013	EPI_ISL_143262	Instituto de Salud Publica de Chile	CDC
A/Peru/114/2013	EPI_ISL_143252	NAMRU-6	CDC
A/Peru/55/2013	EPI_ISL_143251	NAMRU-6	CDC
A/Valdivia/20596/2013	EPI_ISL_142587	Instituto de Salud Publica de Chile	CDC
A/Valparaiso/14542/2013	EPI_ISL_140994	Instituto de Salud Publica de Chile	CDC
A/Buenos Aires/10435982/2012	EPI_ISL_132003	Instituto Nacional de Enfermedades Infecciosas	National Institute for Medical Research
A/Buenos Aires/1004423/2012	EPI_ISL_132002	Instituto Nacional de Enfermedades Infecciosas	National Institute for Medical Research
A/Peru/1026/2012	EPI_ISL_131270	NAMRU-6	CDC
A/Uruguay/06/2012	EPI_ISL_129661	Departamento de Laboratorio de Salud Publica	CDC
A/Peru/1339/2012	EPI_ISL_129405	NAMRU-6	CDC
A/Curico/51369/2012	EPI_ISL_129166	Instituto de Salud Publica de Chile	CDC
A/Belem/119244/2012	EPI_ISL_129163	National Influenza Center	CDC
A/Venezuela/72/2012	EPI_ISL_129162	Instituto Nacional de Higiene "Rafael Rangel"	CDC
A/Guyane/1296/2012 (A/Guadeloupe/44/2012)	EPI_ISL_129053	CRR virus Influenza region Sud	National Institute for Medical Research
A/Paraguay/32/2012	EPI_ISL_129005	Central Laboratory of Public Health	CDC
A/Puerto Montt/51699/2012	EPI_ISL_129001	Instituto de Salud Publica de Chile	CDC
A/Punta Arenas/52090/2012	EPI_ISL_128997	Instituto de Salud Publica de Chile	CDC
A/Vina Del Mar/49586/2012	EPI_ISL_128995	Instituto de Salud Publica de Chile	CDC
A/Santiago/45700/2012	EPI_ISL_128988	Instituto de Salud Publica de Chile	CDC
A/Linares/52087/2012	EPI_ISL_128987	Instituto de Salud Publica de Chile	CDC
A/Suriname/295/2012	EPI_ISL_128986	Caribbean Epidemiology Center	CDC
A/Suriname/297/2012	EPI_ISL_127834	Caribbean Epidemiology Center	CDC
A/Santiago/35234/2012	EPI_ISL_125917	Instituto de Salud Publica de Chile	CDC
A/Santiago/33977/2012	EPI_ISL_125916	Instituto de Salud Publica de Chile	CDC
A/Brazil/7920/2012	EPI_ISL_125910	Instituto Adolfo Lutz	CDC
A/Brazil/8456/2012	EPI_ISL_125906	Instituto Adolfo Lutz	CDC
A/Paraguay/37/2012	EPI_ISL_125905	Central Laboratory of Public Health	CDC
A/Paraguay/146/2012	EPI_ISL_125904	Central Laboratory of Public Health	CDC
A/Brazil/8751/2012	EPI_ISL_125902	Instituto Adolfo Lutz	CDC
A/Santiago/37926/2012	EPI_ISL_124524	Instituto de Salud Publica de Chile	CDC
A/Santiago/37126/2012	EPI_ISL_124523	Instituto de Salud Publica de Chile	CDC
A/Puerto Montt/12477/2012	EPI_ISL_119880	Instituto de Salud Publica de Chile	CDC
A/Santiago/3564/2012	EPI_ISL_119879	Instituto de Salud Publica de Chile	CDC
A/Santiago/14696/2012	EPI_ISL_119711	Instituto de Salud Publica de Chile	CDC
A/Paraguay/726/2011	EPI_ISL_102988	Central Laboratory of Public Health	CDC
A/Paraguay/216/2011	EPI_ISL_101916	Central Laboratory of Public Health	CDC
A/Bolivia/340/2011	EPI_ISL_99074	CENETROP	CDC
A/Bolivia/340/2011	EPI_ISL_99073	CENETROP	CDC
A/Bolivia/340/2011	EPI_ISL_99072	CENETROP	CDC
A/Brazil/1151/2011	EPI_ISL_99068	Instituto Adolfo Lutz	CDC

Strain name (includes country)	Strain ID	Originating laboratory	Submitting laboratory
A/Brazil/1151/2011	EPI_ISL_99067	Instituto Adolfo Lutz	CDC
A/Brazil/1151/2011	EPI_ISL_99066	Instituto Adolfo Lutz	CDC
A/Chile/72/2011	EPI_ISL_99065	Instituto de Salud Publica de Chile	CDC
A/Chile/72/2011	EPI_ISL_99064	Instituto de Salud Publica de Chile	CDC
A/Chile/64/2011	EPI_ISL_99063	Instituto de Salud Publica de Chile	CDC
A/Chile/64/2011	EPI_ISL_99062	Instituto de Salud Publica de Chile	CDC
A/Paraguay/2395/2010	EPI_ISL_99025	Central Laboratory of Public Health	CDC
A/Paraguay/2395/2010	EPI_ISL_99024	Central Laboratory of Public Health	CDC
A/Paraguay/2395/2010	EPI_ISL_99023	Central Laboratory of Public Health	CDC
A/Paraguay/2394/2010	EPI_ISL_99022	Central Laboratory of Public Health	CDC
A/Paraguay/2394/2010	EPI_ISL_99021	Central Laboratory of Public Health	CDC
A/Paraguay/2394/2010	EPI_ISL_99020	Central Laboratory of Public Health	CDC
A/Suriname/5163/2009	EPI_ISL_98967	Caribbean Epidemiology Center	CDC
A/Suriname/5163/2009	EPI_ISL_98966	Caribbean Epidemiology Center	CDC
A/Bolivia/805/2011	EPI_ISL_98825	CENETROP	CDC
A/Santiago/18456/2011	EPI_ISL_98644	Instituto de Salud Publica de Chile	CDC
A/Paraguay/210/2011	EPI_ISL_98643	Central Laboratory of Public Health	CDC
A/Santiago/18454/2011	EPI_ISL_96108	Instituto de Salud Publica de Chile	CDC
A/Santiago/14944/2011	EPI_ISL_96107	Instituto de Salud Publica de Chile	CDC
A/Santiago/13652/2011	EPI_ISL_96106	Instituto de Salud Publica de Chile	CDC
A/Peru/7111/2011	EPI_ISL_96105	NAMRU-6	CDC
A/Peru/6311/2011	EPI_ISL_96104	NAMRU-6	CDC
A/Paraguay/210/2011	EPI_ISL_96101	Central Laboratory of Public Health	CDC
A/Colombia/6459/2011	EPI_ISL_96087	Instituto Nacional de Salud de Columbia	CDC
A/Chile/64/2011	EPI_ISL_96086	Instituto de Salud Publica de Chile	CDC
A/Brazil/6078/2011	EPI_ISL_96085	Instituto Adolfo Lutz	CDC
A/Brazil/5613/2011	EPI_ISL_96084	Instituto Adolfo Lutz	CDC
A/Argentina/8823/2011	EPI_ISL_96080	CEMIC University Hospital	CDC
A/Argentina/676/2011	EPI_ISL_96079	Instituto Nacional de Enfermedades Infecciosas	CDC
A/Argentina/566/2011	EPI_ISL_96078	Instituto Nacional de Enfermedades Infecciosas	CDC
A/Argentina/198/2011	EPI_ISL_96077	Instituto Nacional de Enfermedades Infecciosas	CDC
A/Brazil/6772/2011	EPI_ISL_95509		CDC
A/Argentina/179/2011	EPI_ISL_95507	Instituto Nacional de Enfermedades Infecciosas	CDC
A/Argentina/215/2011	EPI_ISL_95505	Instituto Nacional de Enfermedades Infecciosas	CDC
A/Santa Fe/1431/2011	EPI_ISL_94722	Instituto Nacional de Enfermedades Infecciosas	National Institute for Medical Research
A/Entre Rios755282/2011	EPI_ISL_94721	Instituto Nacional de Enfermedades Infecciosas	National Institute for Medical Research
A/Buenos Aires/10140261/2011	EPI_ISL_94720	Instituto Nacional de Enfermedades Infecciosas	National Institute for Medical Research
A/Chile/9920/2011	EPI_ISL_93777	Instituto de Salud Publica de Chile	CDC
A/Chile/72/2011	EPI_ISL_93776	Instituto de Salud Publica de Chile	CDC
A/Chile/64/2011	EPI_ISL_93775	Instituto de Salud Publica de Chile	CDC
A/Brazil/1151/2011	EPI_ISL_93773	Instituto Adolfo Lutz	CDC
A/Bolivia/405/2011	EPI_ISL_93772	CENETROP	CDC
A/Bolivia/401/2011	EPI_ISL_93771	CENETROP	CDC
A/Bolivia/373/2011	EPI_ISL_93770	CENETROP	CDC
A/Peru/9310/2010	EPI_ISL_89817	NAMRU-6	CDC
A/Peru/8410/2010	EPI_ISL_89816	NAMRU-6	CDC
A/Peru/7710/2010	EPI_ISL_89815	NAMRU-6	CDC
A/Peru/4010/2010	EPI_ISL_89814	NAMRU-6	CDC
A/Argentina/8409/2010	EPI_ISL_87951	Instituto Nacional de Enfermedades Infecciosas	CDC
A/Argentina/02/2010	EPI_ISL_87950	Instituto Nacional de Enfermedades Infecciosas	CDC
A/Argentina/01/2010	EPI_ISL_87949	Instituto Nacional de Enfermedades Infecciosas	CDC
A/Argentina/28379/2010	EPI_ISL_85724	Instituto Nacional de Enfermedades Infecciosas	National Institute for Medical Research
A/Argentina/28378/2010	EPI_ISL_85723	Instituto Nacional de Enfermedades Infecciosas	National Institute for Medical Research
A/Argentina/28372/2010	EPI_ISL_85722	Instituto Nacional de Enfermedades Infecciosas	National Institute for Medical Research
A/Argentina/28370/2010	EPI_ISL_85721	Instituto Nacional de Enfermedades Infecciosas	National Institute for Medical Research
A/Argentina/28367/2010	EPI_ISL_85720	Instituto Nacional de Enfermedades Infecciosas	National Institute for Medical Research
A/Argentina/28342/2010	EPI_ISL_85719	Instituto Nacional de Enfermedades Infecciosas	National Institute for Medical Research

Strain name (includes country)	Strain ID	Originating laboratory	Submitting laboratory
A/Argentina/28306/2010	EPI_ISL_85718	Instituto Nacional de Enfermedades Infecciosas	National Institute for Medical Research
A/Argentina/28302/2010	EPI_ISL_85717	Instituto Nacional de Enfermedades Infecciosas	National Institute for Medical Research
A/Argentina/27724/2010	EPI_ISL_85716	Instituto Nacional de Enfermedades Infecciosas	National Institute for Medical Research
A/Paraguay/2394/2010	EPI_ISL_85613	Central Laboratory of Public Health	CDC
A/Uruguay/2214/2010	EPI_ISL_85601	Departamento de Laboratorio de Salud Publica	CDC
A/Bolivia/1053/2010	EPI_ISL_84043	CENETROP	CDC
A/Argentina/27893/2010	EPI_ISL_83717	Instituto Nacional de Enfermedades Infecciosas	National Institute for Medical Research
A/Argentina/27891/2010	EPI_ISL_83716	Instituto Nacional de Enfermedades Infecciosas	National Institute for Medical Research
A/Argentina/27724/2010	EPI_ISL_83714	Instituto Nacional de Enfermedades Infecciosas	National Institute for Medical Research
A/Argentina/27895/2010	EPI_ISL_83713	Instituto Nacional de Enfermedades Infecciosas	National Institute for Medical Research
A/Argentina/27894/2010	EPI_ISL_83712	Instituto Nacional de Enfermedades Infecciosas	National Institute for Medical Research
A/Venezuela/07/2010	EPI_ISL_83173	Instituto Nacional de Higiene "Rafael Rangel"	CDC
A/Brazil/0791/2010	EPI_ISL_79673	Oswaldo Cruz Foundation - Ministry of Health	CDC
A/Brazil/0610/2010	EPI_ISL_79672	Oswaldo Cruz Foundation - Ministry of Health	CDC
A/Chile/8196/2010	EPI_ISL_79664	Instituto de Salud Publica de Chile	CDC
A/Colombia/6722/2010	EPI_ISL_79660	Instituto Nacional de Salud de Columbia	CDC
A/Chile/6927/2010	EPI_ISL_79330	Instituto de Salud Publica de Chile	CDC
A/Chile/6380/2010	EPI_ISL_79329	Instituto de Salud Publica de Chile	CDC
A/Chile/6278/2010	EPI_ISL_79328	Instituto de Salud Publica de Chile	CDC
A/Chile/6096/2010	EPI_ISL_79327	Instituto de Salud Publica de Chile	CDC
A/Chile/5845/2010	EPI_ISL_79326	Instituto de Salud Publica de Chile	CDC
A/Bolivia/317/2010	EPI_ISL_77796	Instituto Nacional de Laboratoriosde Salud (INLASA)	CDC
A/Colombia/7158/2009	EPI_ISL_76692	Instituto Nacional de Salud de Columbia	CDC
A/Colombia/4335/2009	EPI_ISL_69716		CDC
A/Colombia/6123/2009	EPI_ISL_66567	Instituto Nacional de Salud de Columbia	CDC
A/Bolivia/2948/2009	EPI_ISL_66561	–	CDC
A/Bolivia/2675/2009	EPI_ISL_66560	–	CDC
A/Brazil/884/2009	EPI_ISL_60770	National Influenza Center	CDC
A/Brazil/933/2009	EPI_ISL_60769	National Influenza Center	CDC
A/Brazil/1814/2009	EPI_ISL_60764	Instituto Adolfo Lutz	CDC
A/Argentina/7646/2009	EPI_ISL_60763	Instituto Nacional de Enfermedades Infecciosas	CDC
A/Paraguay/52/2009	EPI_ISL_60745	Central Laboratory of Public Health	CDC
A/Argentina/15/2009	EPI_ISL_34979	–	CDC
A/Suriname/5163/2009	EPI_ISL_34969	Caribbean Epidemiology Center	CDC
A/Venezuela/9602/2007	EPI_ISL_23342	–	CDC
A/Venezuela/8241/2007	EPI_ISL_23341	–	CDC
A/Brazil/1623/2008	EPI_ISL_23203	–	CDC
A/Brazil/1619/2008	EPI_ISL_23202	–	CDC
A/Guyane/32/2007	EPI_ISL_21966	–	CDC
A/Guyane/25/2007	EPI_ISL_21964	–	CDC
A/Guyane/13/2007	EPI_ISL_21962	–	CDC
A/Argentina/449/2007	EPI_ISL_21784	–	CDC
A/Argentina/445/2007	EPI_ISL_21783	–	CDC
A/Argentina/389/2007	EPI_ISL_21781	–	CDC
A/Argentina/3888/2007	EPI_ISL_21780	–	CDC
A/Uruguay/716/2007	EPI_ISL_21292	–	CDC
A/Uruguay/716/2007	EPI_ISL_21291	–	CDC
A/Uruguay/716/2007	EPI_ISL_21290	–	CDC
A/Peru/8307/2007	EPI_ISL_20684	–	CDC
A/Santiago/10086/2007	EPI_ISL_20677	–	CDC
A/Uruguay/0723/2007	EPI_ISL_20638	–	CDC
A/Uruguay/0710/2007	EPI_ISL_20636	–	CDC
A/Uruguay/0707/2007	EPI_ISL_20635	–	CDC
A/Argentina/501/2007	EPI_ISL_20603	–	CDC
A/Argentina/405/2007	EPI_ISL_20602	–	CDC
A/Argentina/426/2007	EPI_ISL_20601	–	CDC
A/Argentina/503/2007	EPI_ISL_20600	–	CDC
A/Argentina/117/2007	EPI_ISL_20599	–	CDC
A/Argentina/335/2007	EPI_ISL_20598	–	CDC
A/Argentina/402/2007	EPI_ISL_20596	–	CDC
A/Argentina/305/2007	EPI_ISL_20595	–	CDC
A/Argentina/146/2007	EPI_ISL_20594	–	CDC
A/Argentina/3797/2007	EPI_ISL_20590	–	CDC

Strain name (includes country)	Strain ID	Originating laboratory	Submitting laboratory
A/Argentina/3743/2007	EPI_ISL_20587	–	CDC
A/Argentina/3726/2007	EPI_ISL_20586	–	CDC
A/Argentina/3689/2007	EPI_ISL_20585	–	CDC
A/Brazil/80/2007	EPI_ISL_20577	–	CDC
A/Peru/3355/2006	EPI_ISL_20573	–	CDC
A/Peru/0128/2006	EPI_ISL_20572	–	CDC
A/Santiago/6881/2007	EPI_ISL_20544	–	CDC
A/Santiago/6421/2007	EPI_ISL_20543	–	CDC
A/Uruguay/716/2007	EPI_ISL_19048	–	CDC

*CDC, Centers for Disease Control and Prevention, Atlanta, GA, USA; NAMRU-6, United States Navy Medical Research Unit-6. The – symbol indicates missing details.
